# An Updated Review of the Epigenetic Mechanism Underlying the Pathogenesis of Age-related Macular Degeneration

**DOI:** 10.14336/AD.2019.1126

**Published:** 2020-10-01

**Authors:** Xiaohua Li, Shikun He, Mingwei Zhao

**Affiliations:** ^1^Henan Provincial People’s Hospital, Zhengzhou, China.; ^2^Henan Eye Hospital, Henan Eye Institute, Henan Key Laboratory of Ophthalmology and Visual Science, Zhengzhou, China.; ^3^People’s Hospital of Zhengzhou University, Zhengzhou, China.; ^4^People’s Hospital of Henan University, Zhengzhou, China.; ^5^Departments of Pathology and Ophthalmology, Keck School of Medicine of the University of Southern California, Los Angeles, CA, USA.; ^6^Ophthalmology Optometry Centre, Peking University People’s Hospital, Beijing Key Laboratory of Diagnosis and Therapy of Retinal and Choroid Diseases, Beijing, China.

**Keywords:** age-related macular degeneration, epigenetics, single nucleotide polymorphisms

## Abstract

Epigenetics has been recognized to play an important role in physiological and pathological processes of the human body. Accumulating evidence has indicated that epigenetic mechanisms contribute to the pathogenesis of age-related macular degeneration (AMD). Although the susceptibility related to genetic variants has been revealed by genome-wide association studies, those genetic variants may predict AMD risk only in certain human populations. Other mechanisms, particularly those involving epigenetic factors, may play an important role in the pathogenesis of AMD. Therefore, we briefly summarize the most recent reports related to such epigenetic mechanisms, including DNA methylation, histone modification, and non-coding RNA, and the interplay of genetic and epigenetic factors in the pathogenesis of AMD.

Age-related macular degeneration (AMD) is the leading cause of irreversible blindness in the elderly in developed countries. AMD can manifest as either geographic atrophy (GA) in the dry form or choroidal neovascularization (neovascular AMD, nAMD) in the wet form of AMD [1]. there are multiple, complicated risk factors for AMD [2]. The incidence of AMD increases with age and the condition afflicts millions of adults worldwide. Although it is well-recognized that genetic factors, retinal cell senescence, and an abnormal environment such as smoking, UV light or serum lipid status is involved in the initiation of AMD, the pathogenesis of AMD remains unclear [3]. In the last decade, accumulating evidence has suggested that epigenetics may play an essential role in the pathogenesis of AMD.

“Epigenetics” refers to the modifications of DNA by external factors, which turn genes “on” or “off,” resulting in changed functions and behaviors of an organism. These modifications do not cause changes to the DNA sequence, but they are heritable, dynamic, and reversible. Epigenetics include DNA methylation, histone modification, non-coding RNA, RNA methylation, and so on. Since the 1990s, with advances in epigenetic research, several new concepts and terms, such as the epigenome, epigenetic epidemiology, epigenetic pathology, epigenetic disease, epimutation and epigenetic therapy, have been introduced.

It is well-known that aging, smoking, sun exposure, lack of a balanced diet, and oxidative stress are common risk factors for AMD; however, how these risk factors contribute to the pathogenesis of AMD remains mostly unexplored. An updated study of AMD etiology suggested that the influence of these risk factors are accumulated over the whole lifespan, which may trigger aalteration of epigenetic factors, such as DNA methylation, histone modification, and non-coding RNA, which may then influence the pathogenesis of AMD by changing gene expression. Although the extent of epigenetic change contribution to AMD remains to be clarified, understanding the role of epigenetics in its pathogenesis is important, since treatment of the disease is based on an understanding of its pathogenesis. Demonstrating a role for epigenetic mechanisms in AMD may provide a new understanding of the disease pathogenesis, and regulation of epigenetic factors may provide a potential therapeutic approach for treating this complex disease. In this review, we briefly summarized the most recent progress in the study of the relevance of epigenetics to AMD.

## The role of susceptibility genes in AMD pathogenesis

Previous studies have shown that alteration of multiple genes may be associated with the susceptibility to AMD; the genetic abnormalities in patients with AMD are more likely to involve single nucleotide polymorphisms (SNPs), in which one nucleotide within a gene is substituted by another. To date, numerous SNPs have been found in genes from samples of patients with AMD, including APOE, CFH, HTRA1, TLR3, TLR4, LIPC, C3, C2/CFB, VEGFA, ABCA4, ERCC6, CX3CR1, TNFRSF10A [4-6], COL8A1/FILIP1L, IER3/DDR1, SLC16A8, TGFBR1, RAD51B, ADAMTS9/MIR548A2, B3GALTL, and ARMD1 [7].

It is known that the most prevalent SNPs associated with AMD occur in CFH and HTRA1 [8-10]. A study reported by Millen et al. shows that the risk of AMD is higher in individuals with vitamin D deficiency and SNPs in CFH, suggesting a possible synergistic effect of vitamin D deficiency and variations in CFH [11]. Vladan et al. indicated that SNPs in *DIAPH2*, located on the X-chromosome, are also associated with susceptibility to AMD [12].Notably, the contribution of SNPs in the risk of development of nAMD varies across different populations [13]. In addition, the outcomes of intravitreous injection of anti-VEGF reagents in the treatment of CNV are influenced by an individual’s genetic background. Furthermore, the genetic variants in *IL17A* are functionally associated with an increased risk of AMD [14].

Polypoidal choroidal vasculopathy (PCV) and CNV are the most common types of nAMD. The prevalence of PCV is higher in Asian populations than in Caucasian populations. The association of genetic risk factors of CFH, HTRA1, and FPR1 in CNV and PCV is similar [15, 16], but other SNPs, such as those in SKIV2L, are seen only in patients with CNV, but not in those with PCV [17]. In contrast, in patients with PCV, the rs5882(GG) SNP in CETP has a markedly higher frequency than that in patients with CNV [18]. Furthermore, Ji et al. found that SNP rs6982567 in GDF6 may serve as an additional risk factor of PCV in the Chinese population [19]. Interestingly, the risk factors may be variable in different phenotypes of PCV, as a report by Yanagisawa et al. showed that rs868005 in ELN was significantly associated with type 2 PCV, abnormal ELN may alter the structure and function of elastin, suggesting the importance of elastin in the pathogenesis of PCV and the possibility of using the *ELN* SNP as a marker for the differentiation of PCV phenotypes [20]. Taken together, these studies imply that genetic background contributes to the pathogenesis of AMD.

## Role of epigenetic mechanisms in AMD pathogenesis

The susceptibility to AMD conferred by genetic variants has been revealed by genome-wide association studies, as mentioned above; these genes may predict the risk of AMD in less than half of the human population [21, 22]. Seddon et al. claimed that although 46-71% severity of AMD may be attributed to genetic background, the remaining 19-37% cases may be influenced by epigenetic factors [23] The pathogenesis of AMD, a complex disease, cannot be entirely explained by genetic variations; if the pathogenesis of AMD is solely controlled by genetic factors, theoretically, the disease should occur early rather than late in life. Importantly, epidemiological studies have demonstrated that advanced AMD in monozygotic twin is more likely to be associated with the habit of heavy smoking and reduced levels of vitamin D, betaine, and methionine uptake; all these environmental factors may contribute to the pathogenesis of AMD through epigenetic factors [23]. Therefore, the study of the relevance of epigenetics in the occurrence of AMD may provide new understanding of the pathogenesis of and therapeutic approach to AMD.

## Relevance of DNA methylation to AMD

The first evidence to show the relevance of DNA methylation to AMD is the abnormal methylation of CLU and gene encoding clusterin, which contains CpG islands in its promoter region and which may have anti-inflammatory and anti-angiogenic functions. Interestingly, treatment of ARPE-19 cells with the DNA methylation inhibitor 5-azacytidine (5-AZA) upregulated CLU expression, suggesting that its expression is subject to the regulation of DNA methylation [24]. Later, in a study involving mapping of promoter DNA methylation in AMD and age-matched normal retinal pigment epithelium/choroid samples, it was found that the genes encoding antioxidants glutathione S-transferase isoforms mu1 and mu2 are heavily methylated in their promoter regions and downregulated in AMD samples [25]. Additionally, reduced methylation of the promoter of the pro-angiogenic ANGPTL2 was confirmed in AMD samples [25]. Hypomethylation of the IL-17RC promoter has recently been identified in peripheral blood cells from patients with AMD and was associated with increased expression of this gene in the peripheral blood and may affect the inflammatory response in the retina and choroid. The study suggested that abnormal expression of IL-17RC, regulated by epigenetic factors, may play a role in the pathogenesis of AMD [25]. However, a report by Oliver *et al.* [26] showed that there was no significant difference in the methylation of the IL17RC promoter in AMD patients compared to control subjects, suggesting that further studies are necessary regarding the use of IL-17RC promoter methylation as a biomarker of AMD.

Among many environmental risk factors, smoking is the top factor increasing the risk of AMD development. Importantly, the report by Seddon et al. showed that a heavier-smoking twin tended to have more advanced stage AMD, associated with larger drusen and pigmentation [23]. In contrast, smaller drusen and less pigmentation in the fundus was correlated with an earlier stage of AMD in twins [23]. The results imply that individual lifestyle factors play a role in the pathogenesis of AMD through epigenetic mechanisms [23]. Direct evidence of the association of smoking with epigenetic changes was confirmed by Koks et al. [27], who found that methylation of GPR15 was significantly lower among non-smokers; the reduced methylation of GPR15 leads to up-regulation of this gene and a chronic inflammatory response. The study explained the relevance of smoking and DNA methylation in the pathogenesis of AMD in detail [27].

Recently, a report by Oliver et al. showed that no obvious alteration of methylation sites was revealed by a genome-wide DNA methylation analysis of blood samples from AMD patients and controls; however, a significant DNA methylation difference in the position close to ARMS-2 was found in the peripheral blood of nAMD patients [26]. Furthermore, abnormal DNA methylation was demonstrated in the promoter region of PRSS50 from retinal and blood samples of AMD patients [28]. Importantly, the methylation status of VEGF (one of the key growth factors in the induction of nAMD) is signiﬁcantly di?erent in AMD patients compared with control subjects [29]; the results provide a new clue as to why VEGF expression is increased in nAMD patients.

All the above reports about DNA methylation relevant to AMD focused on changes in individual genes and lacked genome-wide DNA methylation data. Recently, an interesting study by Louise F et al. demonstrated that there was differential DNA methylation of SKI, GTF2H4, and TNXB as analyzed by genome-wide DNA methylation in human RPE cells obtained from AMD patients [30]. The most significant point of their study was that the alteration in DNA methylation was not related to global aberrant DNA methylation but was observed in local tissue. These findings imply that DNA methylation in the specific tissue may be of more importance than global DNA methylation in the understanding of AMD pathogenesis.

The finding of altered DNA methylation in AMD risk genes suggest that epigenetic mechanisms contribute to the pathogenesis of AMD and that DNA methylation may serve as an additional type of epigenetic marker for the prediction of AMD risk [28].

## Relevance of histone modification to AMD

**Figure 1.**
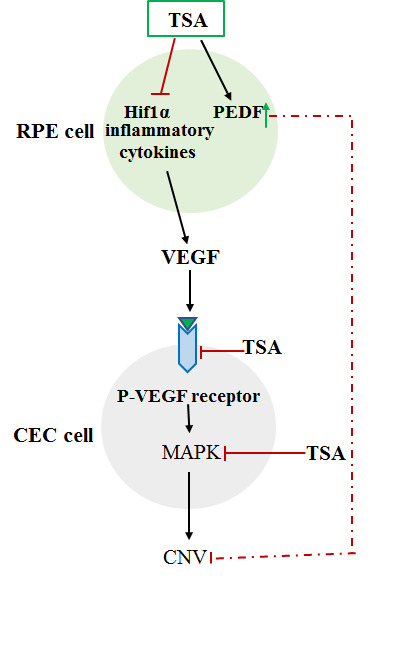
Inhibition of CNV by TSA may involve multiple mechanisms: (1) Suppression of HIF1α and inflammatory cytokines as well as VEGF expression; (2) Inhibition of the phosphorylation of VEGF receptor 2 induced by VEGF; (3) Inhibition of activation of MAPK; (4) Upregulation of the expression of the anti-angiogenesis factor PEDF. CNV, choroidal neovascularization; TSA, trichostatin A.

Hypoxia-inducible factor-1α (HIF1α), whose downstream target genes include VEGF, has been suggested to contribute to the pathogenesis of nAMD [31]. Epigenetic regulation of HIF1α has been evaluated in cell culture and cancer models. The expression of HIF1α is reduced via application of histone deacetylase inhibitor (HDACi), which also inhibits the expression of VEGF [32]. In RPE cells, hypoxia increases the expression of histone lysine demethylase (histone lysine demethylases, KDMs), thereby promoting the expression of pro-angiogenic genes, such as ADM, GDF15, HMOX1, SERPE1, and SERPB8; however, the KDM-encoding genes and JMJD1A are able to inhibit the expression of these genes induced by hypoxia and can lead to suppression of angiogenesis [32]. Another report showed that reduction of HDAC7 expression is associated with decreased transactivation of HIF1α [33], but VEGF induces the nuclear egress of HDAC7 and can activate pro-angiogenic gene expression; therefore, inhibition of HDAC7 expression may inhibit VEGF-induced angiogenesis [34]. Trichostatin A (TSA) is a HDACi that inhibits the activity of classes I and II HDACs [35]. TSA and other HDACis could not only inhibit endothelial cell proliferation but also downregulate VEGF-receptor expression [36-38]. Notably, a patent has been filed for the application of HDACi in the treatment of ocular neovascularization or edema-related diseases, including AMD and diabetic retinopathy [39]. Furthermore, as additional evidence supporting the role of histone modifications relevant to AMD pathogenesis, Gnana-Prakasam et al. reported significant increases in mRNA expression of HDAC1, HDAC3, HDAC6 in mouse RPE cells associated with excessive iron levels, which may be considered as one of the risk factors for AMD [40]. The importance of histone acetylation/deacetylation was further validated by Chan et al., who demonstrated that the pro-angiogenic HIF1α and VEGF are downregulated in RPE cells treated with TSA [41]. Most interestingly, the anti-angiogenic neuroprotective pigment epithelium derived factor (PEDF) is highly expressed in RPE cells treated with TSA [41]. Furthermore, as the key receptor in the mediation of angiogenesis, VEGFR2 expression in human vascular endothelial cells is significantly suppressed by TSA treatment [41]. Notably, the wound-healing response in the process of CNV formation is also inhibited by TSA treatment in vivo, as shown by the reduction of smooth muscle actin expression [41]. One of the mechanisms underlying the inhibition of CNV by TSA treatment involves inactivation of the MAPK pathway, thereby inhibiting endothelial cell proliferation, migration, and tube formation [41]. In an ischemic rat retina model, TSA not only protected the retina from ischemic damage, but also inhibited the induction of matrix metalloproteinase-1 and matrix metallo-proteinase-3 expression by TNF-α [42]. Most importantly, CNV formation in animal models is inhibited by TSA application [41], suggesting that TSA application is a potential therapeutic approach for the treatment of CNV (Fig. 1).

**Figure 2.**
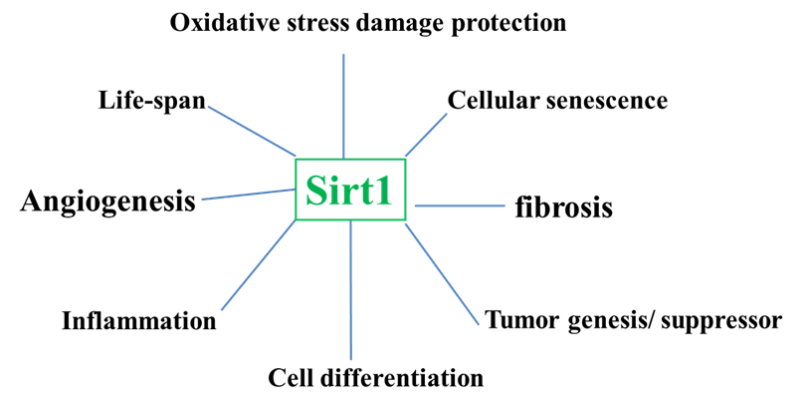
Function of SIRT1. Biological functions of Sirt1 are broad, ranging from aging, anti-oxidative protection of cells, inhibition of inflammation, angiogenesis, and fibrosis, and tumorigenesis suppression.

A further hotspot in the study of histone acetylation/deacetylation relevant to AMD is SIRT1, encoding NAD-dependent deacetylase sirtuin-1, a histone deacetylase converting enzyme [43]. Cell senescence, DNA damage repair, apoptosis, inflammation, and angiogenesis are regulated by SIRT1 [43-46] [Fig. 2]. SIRT1 not only targets deacetylated histones, but also regulates a variety of other non-histone proteins, such as P53 [45-47], FOXO [48], NF-κB [49], E2F1 [50], PGC-1α [51], HIF-1 [52], and HIF-2 [53]. Therefore, SIRT1 plays an important role in maintaining normal cellular functions, and its abnormal expression may be related to pathological conditions.

The expression of *SIRT1* was significantly lower in RPE cells obtained from AMD patients than in those obtained from age-matched controls [54]. However, as increased SIRT1 expression was seen in human CNV membranes compared with non-AMD donor eyes [55]; the variable SIRT1 expression may reflect the differences in AMD stage.

**Figure 3.**
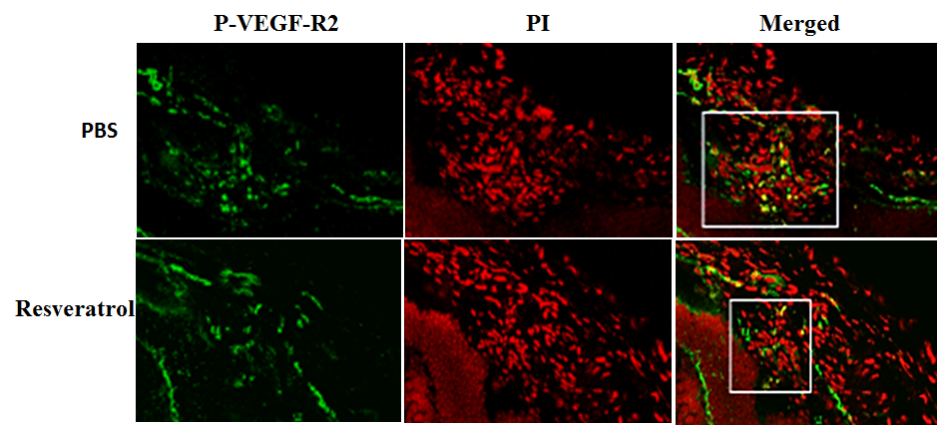
Resveratrol inhibits experimental CNV.Resveratrol inhibits VEGF receptor 2 phosphorylation (P-VEGF-R2) in mice with a choroidal neovascular (CNV) lesion induced by laser, as showed by immunofluorescence. Green indicates positive staining of P-VEGF-R2 and red is propidium iodide (PI) staining. Yellow represents double labeling. the P-VEGF-R2 staining is considerably reduced in mice with a CNV lesion after resveratrol application (square). Original magnification ×400.

SIRT1 protects RPE cells from cell senescence and apoptosis induced by oxidative stress. Fullerenol and vitamin C-mediated RPE protection is also thought to be mediated through activation of SIRT1 [56, 57].SIRT1 may exert its cytoprotective effect through various signaling pathways, including deacetylation of P53, activation of ERK1/2, phosphorylation of NRF-2, increasing expression of the antioxidant-response genes HO1 and NQO1, [58]. The beneficial effects of oral supplementation of SIRT1 activator-resveratrol (RSV) have been reported in AMD patients [59]; this is similar to the results obtained from a recent clinical trial on Alzheimer’s disease [60]. Our study showed that application of the SIRT1 activator RSV inhibited VEGF-induced endothelial cell migration and tube formation. The HIF1α accumulation and VEGF secretion induced by cobalt chloride (CoCl_2_) are inhibited by RSV treatment in human RPE cells [61]. Furthermore, we demonstrated that VEGFR2 phosphorylation in a laser-induced CNV lesion is downregulated by RSV treatment [Fig. 3]. Most importantly, laser-induced CNV formation is significantly inhibited by intravitreal injection of RSV [61]. These results suggest that the inhibitory effect of RSV on CNV is mediated through the HIF1α/VEGF/VEGFR2 signaling axis [61], and that SIRT1 may protect RPE against oxidative stress-induced damage and can be used as an adjuvant or combination therapy for AMD.

## Relevance of non-coding RNA to AMD

One essential component of the non-coding RNA family is miRNA. By transcriptome analysis of the miRNome, it was found that 480 miRNAs are expressed in the human retina and 416 miRNAs are expressed in the RPE/choroid in healthy individuals [62]. The relevance of miRNA to the pathogenesis of AMD has attracted marked attention. miR-15, miR-16, miR-20a, and miR-20b are able to inhibit VEGF mRNA expression, while the miR-17-92 cluster, miR-27b, let-7f, and miRNA-107 can induce angiogenesis [63] by targeting HIF1α and *VEGF* [64]. Downregulation of miR-23a results in RPE cell apoptosis [65]. Interestingly, a number of inflammatory cytokines, such as TNF-α, IL-1β, and interferon-γ, could induce expression of miR-155 through the Janus kinase pathway. Upregulated expressions of miRNA-9, miRNA-125b, miRNA-146a, and miRNA-155 are found in the aged retina; all these are responsive to NF-κB activation [66]. CFH SNPs associated with AMD are well documented; interestingly, CFH expression can be regulated by various miRNAs, such as miR-9, miR-125b, miR-146a, and miR-155 [64].

The importance of miRNA in the pathogenesis of AMD has also been confirmed in an animal experiment. It was shown that CNV volume is significantly suppressed in a laser-induced murine model of CNV by intravitreal injection of pre-miR-21 [67]. Under ischemic conditions, application of pre-miR-31 or pre-miR-150 could inhibit the expression of VEGF in the retina; in addition, the expression of retinal HIF1α and *PDGFB* in the retina is compromised by pre-miR-31. DICER1, an important miRNA-processing enzyme, is significantly reduced in samples from human patients with dry AMD. Deletion of DICER1 results in the accumulation of Alu RNA and leads to RPE death. An RPE degeneration phenotype is displayed in a mouse model with a DICER-knockdown in the retina [68]. Saxena found that intense light exposure for 24 hours led to upregulation of 19 miRNAs, some of which are related to the inflammatory immune response or which trigger retinal damage associated with AMD [69].

A study by Strafella et al. showed that SNPs in genes encoding miR146a and miR27a may serve as biomarkers of susceptibility to AMD, after screening of 976 nAMD patients and 1000 control subjects using epigenotyping analysis [70]. Their findings suggested that both miR27a and miR146a were involved in angiogenesis and inflammation in the pathogenesis of nAMD.

Current miRNA studies focus on the circulating miRNAs in the blood; circulating miRNAs could be used as functional biomarkers for the diagnosis of human diseases, while miRNAs incorporated into polymer-based hydrogel nanoparticles may be used for the treatment of AMD [71]. Recently, it has been reported that miRNA may be considered as a potential biomarker of AMD: Grassmann et al. found that the blood has-miR-424-5p level is significantly elevated in dry AMD compared to nAMD patients [72]. Another study showed that the expression of miR-661 and miR-3121 is elevated in the serum of patients with dry AMD, while increased expression of miR-4258, miR-889, and let-7 is seen in patients with nAMD [73]. Notably, the importance of circulating miRNA to the pathogenesis of AMD is further supported by the study of Ren et al., which demonstrated that the level of a cluster of miRNAs, including miR-27a-3p, miR-29b-3p, and miR-195-5p, was increased in AMD patients, suggesting that they could be potential biomarkers for AMD [74].

In addition to circulating miRNA, the miRNA profile from the vitreous of AMD patients has been reported [75]; a significantly increased expression of miR-146a was revealed in AMD patients, while in contrast, the expression of miR-106b and miR-152 is reduced in the vitreous humor of nAMD patients. Similarly, downregulation of miR-152, which regulates the expression of VEGF, was shown in the vitreous and blood of AMD patients [64]. The profile of expression of miRNA in local tissue may provide more meaningful information about the role of miRNA in AMD pathogenesis.

Zhu et al. reported that long noncoding RNAs (lncRNA, RP11-234O6.2) may be involved in the pathogenesis of early AMD [76]. Their study found that the lncRNA was downregulated in RPE cells by H_2_O_2_ treatment and that overexpression of RP11-234O6.2 was able to inhibit RPE cell apoptosis. The relevance of other lncRNA ZNF503-AS1 to AMD was also revealed by analysis of AMD patient samples [79]. piRNAs, another hot point of noncoding RNA research, was shown to play a role in the pathogenesis of AMD; up to 102 piRNAs are detected by RNA-seq analysis in human RPE cells, and importantly some piRNAs are increased upon oxidative stress in RPE cells. Because oxidative damage contributes to both the onset and progression of AMD, the potential role of interaction of non-coding RNA with oxidative stress in the pathogenesis of AMD should be further addressed [78].

**Figure 4.**
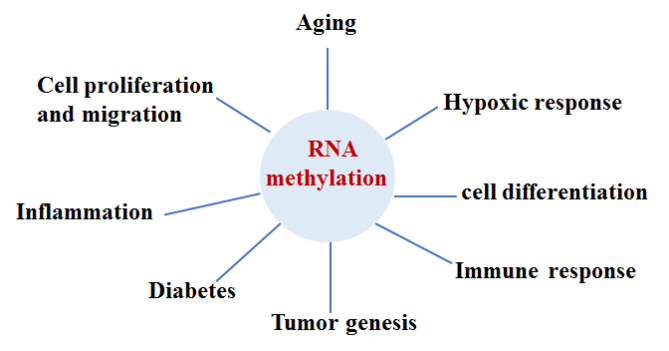
Relevance of RNA methylation to cellular processes. RNA methylation may impact various cellular processes, including cell differentiation, proliferation, migration metabolism, and regulation of inflammation, the immune response, hypoxia, and tumorigenesis.

## Potential role of RNA methylation in AMD pathogenesis

RNA methylation is a new hot topic in the study of epigenetic modification. RNA can be methylated at the N 6-methyladenosine (m^6^A), N 1-methyladenosine (m1A), and 5-methylcytosine (5-mC). m^6^A is the most common and abundant type of RNA methylation and 80% of all RNA methylation occurs at m^6^A. Similar to DNA methylation, the m^6^A methylation is also reversible and dynamic; RNA methylation and demethylation are controlled by its methyltransferases (“writers”), binding proteins (“readers”), and demethylases (“erasers”) [79, 80]. Although intracellular RNA undergoes many modifications, the current research on RNA methylation is primarily focused on m^6^A. Loss of balance between m6A methylation and demethylation can affect physiological functions and even lead to a series of pathological conditions. Reports on RNA methylation showed that m^6^A and 5-mC RNA methylation affects a variety of biological processes, particularly post-translation modification, and aberrant RNA methylation, linked to pathological conditions, such as aging, immune response, hypoxic stress response, tumorigenesis, neurodegeneration, and infection diseases [81-83]. Interestingly, low expression of m^6^A is considered to be a novel potential biomarker of type 2 diabetes [84]. m^6^A is also demonstrated to be a regulator of proliferation, apoptosis, development of therapeutic resistance, and tumorigenesis [80] [Fig. 4]. Latest research shows that RNA methylation could be regulated by histone modification [85, 86]. With the application of m^6^A sequencing technology, studies of m^6^A levels, its distribution in the intracellular transcriptome, and its relevance to cellular functions have been greatly promoted. However, because m^6^A methylation is jointly regulated by methyltransferase, demethylase, and m^6^A recognition protein, its regulatory function in the human body is a complex and dynamic process, and therefore, the phenotypes of a cell may be representing the effects of combinations of various epigenetic factors. Recognition of the importance of RNA methylation in the study of epigenetic research is only the start of such research. Numerous problems remain to be solved; for example, at present, the biggest challenge in the study of m^6^A modification is the lack of dynamic real-time monitoring of m^6^A changes physiologically and pathologically, how the cell selects specific mRNAs for methylation, whether other proteins are involved in the pathogenesis of certainly diseases, interplays between RNA methylation and other epigenetic factors, and normal levels of RNA methylation. AMD has many pathological features, as other systemic diseases, such as oxidative stress damage, inflammation, senescence, cell death, and abnormal cell proliferation and migration in the RPE. Thus, RNA methylation may be a new research direction for studies of the etiology of AMD and biomarker searching for early diagnosis of AMD.

## Potential role of interplays of genetic and epigenetic factors in AMD pathogenesis

Genetic variants affect DNA methylation; SNPs also influence the levels of methylation at CpG sites. AMD is a complicated disease mediated by multiple factors, including genetic, epigenetic, and environmental factors. Much attention has been paid to the contribution of genetics and epigenetics to AMD pathogenesis; however, how the interplay of genetic and epigenetic factors affects the development of AMD remains unclear.

Collective evidence from the study of systemic diseases has shown that genetic and epigenetic factors could affect each other and trigger different types of pathogenesis processes. Genetic variations often reflect the nature of the chromatin structure that is modified by epigenetic factors. On the other hand, epigenetic aberrations also affect genetic stability and gene expression. Therefore, the interaction of genetic and epigenetic alterations determines the pathogenesis and the outcome of a disease.

SNPs are a type of genetic variation and have been linked to numerous pathological conditions. Frank-Bertoncelj et al. found that 31 SNPs associated with rheumatoid arthritis were involved with histone modification of H3K4me3 in CD4^+^ T regulatory cells and that alteration of histone H3K4me3 was also correlated with 67 SNPs in pancreatic and liver cells of type 2 diabetes patients [87]. On the other hand, lncRNAs, such as HOTAIR, can affect gene expression by modification of the chromatin structure [88]. Clearly SNPs and epigenetic factors may affect each other,but the mechanism of how SNPs alter gene expression through epigenetic mechanisms is still under investigation. It may be mediated through the regulation of non-coding RNA and then affect expression of genes, including distant target genes; epigenetic factors that interact with the chromatin may be affected by SNPs, and therefore influence cellular function [87]. The importance of the association of genetic variants, such as SNPs, with epigenetic factors in the contribution to diabetes, ulcerative colitis, and cancer has been revealed [89, 90]. The notion is further supported by studies of liver tumors [91-93]. Analysis of whole genome sequencing combined with epigenome sequencing have identified that liver oncogenes are mutated by genetic or epigenetic alterations; the significance of epigenetic alteration in the development of liver cancer has been demonstrated [92]. DNA methylation is the best studied epigenetic mechanism in systemic disease, as well as AMD. DNA methylation can prevent transcriptional factors from binding to a gene; often methyl-CpG-binding domain proteins may bind to methylated DNA and recruit HDAC1 and SIN3, to inactive chromatin [94]. DNA methylation can affect chromosomal integrity and instability, therefore affecting gene expression. In addition, it has been found that DNA methylation of the region containing BRD2 and HLA is associated with SNPs in liver cancer [91], suggesting that DNA methylation and genetic variation may interact with each other.

Chromatin can respond to external input through many ways, including histone modification, which plays a key role in chromatin remodeling [95]. Histone modification changes chromatin structure by recruitment of remodeling enzymes and forming a complex to influence cellular function. Abnormal histone modification may affect chromatin structures by which gene expression is regulated, and so contributes to many pathological processes in humans. Accumulating evidence indicates that genetic and epigenetic mechanisms form a convoluted network with interactions in both physiological and pathological conditions.

In the retina, 34 AMD-associated SNPs were found by the International AMD Genomics Consortium [96]. The retina is rich in non-coding RNA; a report recently showed that there are 3,582 non-coding RNAs in the peripheral retina of AMD specimens and 3,210 in the RPE-choroid-sclera of the AMD retina [97]. The association of non-coding RNA SNPs rs11671784 (MIR27A, G/A) and rs2910164 (MIR146A, C/G) with AMD has also been reported [70]. This suggests that there may be an interplay between miRNA-related variants and AMD. However, few reports have shown the interplay of AMD-related SNPs with non-coding RNA in the retina or AMD specimens; therefore, much more research is needed to address these essential questions.

## Potential of epigenetic therapy in the treatment of AMD

The most extensive study of the relevance of epigenetics to diseases and epigenetic therapy has focused on cancer [98, 99]. Certainly, epigenetic markers might be used as alternative biomarkers for diagnosis and treatment of certain cancers and other systemic diseases. Currently, the reagents involved in epigenetic therapies may include the DNA methyltransferase inhibitors 5-azanucleosides, azacitidine decitabine, and zebularine, the histone deacetylase inhibitors vorinostat and romidepsin, TSA and suberoylanilide hydroxamic acid (SAHA), the manipulation of non-coding RNA, and the regulation of histone methylation and deacetylation using small molecules [29].

Currently, there is no cure for AMD. The most common treatment method of nAMD is the injection of anti-VEGF reagents; however, complications are seen within the eye and systemically. The variability of individual responses to the injection and the requirement for repeated injections of anti-VEGF reagents are additional challenges. As discussed above, various aberrant epigenetic factors that contribute to or are associated with AMD have been demonstrated in the last decade. Further progress in understanding the role of epigenetics in the pathogenesis of AMD may lead to the development of potential therapies for the treatment of AMD [100].

Below, we discuss the categories of the current research on epigenetic therapy.

## Potential target for DNA methylation in the treatment of AMD

Inhibition of DNA methylation is one of the common approaches to regulate DNA methylation, and thereby activate or inhibit certain genes. The most common application of DNA methylation inhibition is seen in the treatment of tumors; the notion of the value of using DNA methylation inhibitor in treating AMD was demonstrated by the study of 5-aza-2′-deoxycytidine (5-AZA-dc) in the upregulation of clusterin expression in RPE, suggesting that there is hypermethylation in the promoter region of*CLU* [24]. Hunter et al. found that promoter methylation of the glutathione S-transferase isoforms mu1 and mu5 underwent epigenetic repression in human AMD specimens, suggesting that DNA methylation inhibition may be a potential treatment option for AMD [24]. On the contrary, there was a low level of methylation of the *IL17R* in samples from AMD patients. Reduced DNA methylation in the promoter of *LINE1* was revealed, which could be restored by resveratrol in human RPE cells [101], suggesting that either inhibition or enhancement of DNA methylation could be a new strategy for the treatment of early AMD according to the individual condition. In addition, we treated the polarized RPE monolayer cultures with up to 6 µM 5-AZA-dC for 4 days; no detectable morphological changes were found in hematoxylin and eosin staining. The expressions of ZO-1 and cytokeratin, transepithelial resistance, and cell death were analyzed; no obvious abnormality was identified [102]. Theoretically, the use of 5-AZA-dc may be feasible in AMD treatment, because it is a DNA methylation inhibitor approved by the FDA for clinical trial in the treatment of cancer.

## Potential HDAC targets in the treatment of AMD

Loss of the balance of histone acetylation and deacetylation is linked to many pathologic conditions, such as inflammation, cell death, aging, fibrosis, and angiogenesis. Some HDACis, a novel epigenetic therapeutic drug, are now approved by the FDA for the treatment of cancer; therefore, HDAC inhibition by HDACi has been studied extensively in the treatment of various systemic and eye diseases [42, 103-105]. There are various HDACis, including vorinostat, panobinostat, belinostat, ITF2357, PCI-24781, FK228, entinostat, MGCD0103, phenyl butyrate, valproic acid, trichostatin A, LAQ824, mocetinostat, and pracinostat, which are currently under investigation for possible therapeutic purposes [106]. Most of the HDACis have a broad spectrum of HDAC inhibition; only a few HDACis target relatively specific HDACs; the important point is that some HDACis not only inhibit HDAC activity, but also target many non-histone proteins. Therefore, HDACi application is being studied for the treatment of a wide variety of human diseases, including eye diseases [61, 107-111]. One example is SIRT1 (class III HDAC); as mentioned previously, stimulation of SIRT1 by resveratrol significantly promoted GFAP, anti-angiogenic PEDF, and TSP-1 expression in the cells as well as in the phagocytic activities [111].

TSA is an HDACi; in the study of inhibition of CNV, TSA was shown to be a promising HDACi for the inhibition of experimental laser-induced CNV in mouse. The study found that TSA can suppress inflammatory cytokine production, choroidal endothelial cell proliferation, and migration, by downregulating HIF1α, VEGF [111], ANG2, and endothelial cell NOS [100], and upregulating PEDF [111] expression. Most importantly, the expression of VEGFR2 was inhibited by TSA in human vascular endothelial cells; thereby attenuating neovascularization. Injection of TSA could also protect the rat retina from ischemia-induced damage by suppressing TNFα expression in the retina [42]. Using HDACis to inhibit CNV formation was further validated by a study of resveratrol, a SIRT1 activator, reported by our research group [61] and others [112]. Resveratrol can be injected systemically or even intravitreally, to suppress CNV, and also inhibits VEGFR-2 phosphorylation in vivo [61]. Interestingly, a natural compound, sulforaphane (SF), an isothiocyanate, has anti-oxidative and anti-inflammatory effects and inhibits HDACs; it may be a potential epigenetic therapeutic agent [113].

Besides DNA methylation inhibitors and HDACis, some other reagents may hold promise as potential epigenetic therapies. These include vitamin A, which is able to regulate thrombospondin and PEDF in human RPE cells by altering the chromatin structure [114].

**Figure 5.**
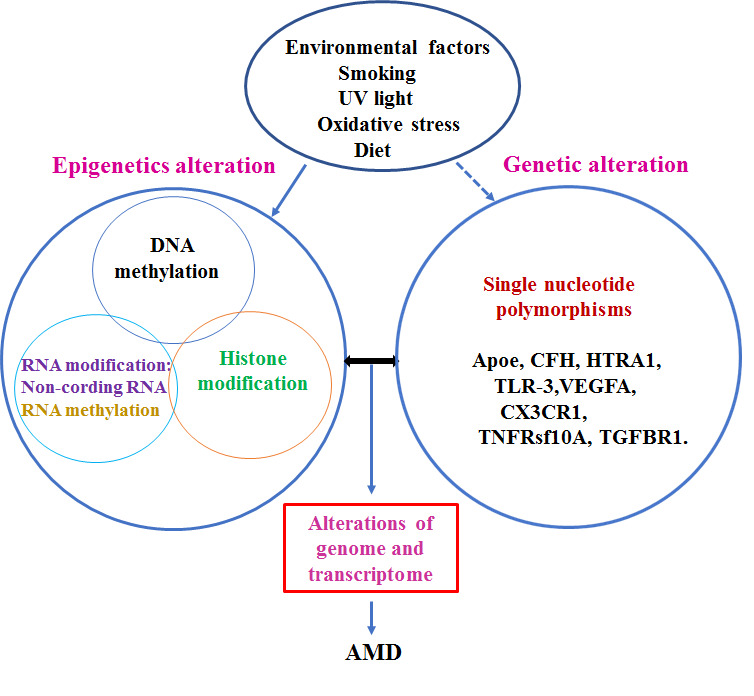
The role of epigenetic factors in the pathogenesis of AMD. Interaction among environmental, genetic, and epigenetic factors may determine initiation of the pathogenesis of AMD. Environmental factors may contribute to the alteration of epigenetic factors, changes in epigenetic mechanisms may induce abnormal gene expression through crosstalk with the genome. On the other hand, genetic alterations, such as single nucleotide polymorphisms, may affect epigenetic factors. Furthermore, there is an interplay among epigenetic regulation pathways, suggesting that AMD is a complex disease. An aberration of genome transcription that is related to AMD is regulated by both genetic and epigenetic factors.

### Prospective studies

With the expansion of epigenetic research, several new concepts and terms have emerged, such as the EWAS, epigenome, epigenetic epidemiology, epigenetic pathology, epigenetic disease, epimutation, and epigenomic therapy [115]. However, these categories are still under investigation, particularly in eye diseases, such as AMD. Although much progress has been made, there are still many challenges. For example, much attention is paid to the status of DNA methylation in gene promoters, but not much information is available on non-promoter-associated modifications, including DNA methylation, noncoding RNA, chromatin architecture, and RNA methylation. In addition, there is no report on the relevance of RNA methylation in AMD and there is no normal standard of DNA methylation and analysis of DNA methylation at a single-cell level in AMD patients or animal models. Because epigenetic modification is dynamic and individual-specific, further research is needed in these directions.

In term of AMD risk factors, besides known SNPs and environmental factors, epigenetic factors may be an additional biomarker through application of a high-throughput sequencing technique, which may be a new approach to predict the possibility of AMD development and facilitate provisional treatment. Tissue-specific changes in epigenetic factors in the RPE and other cells involved in the pathogenesis of AMD are critical when searching for epigenetic markers of AMD. Combinations of epigenetic factor changes in the blood and retinal-specific tissue or cells and SNPs may allow identification of biomarkers of AMD [87].

Detecting the epigenetic abnormality in all known AMD rick loci in AMD patients and age-matched control subjects will be future challenges but could provide answers to some key questions regarding the relevance of epigenetics to AMD pathogenesis. Alleles variants in all introns and exons of relevant genes should be analyzed by EWAS, to establish the distribution of DNA methylation of those genes in the context of the entire nucleotide genome, to identify variations associated with AMD in a large study sample. In addition, identifying the readers of histone modifications is also important for studying the role of histone modification in AMD [116]. RPE, choroidal endothelial cells, and even photoreceptor cells may be included in this type of study. The database will certainly be valuable to localize epigenetic markers of AMD risk, predict responses and/or adverse effects, to facilitate the design of improved epigenetic-based agents and optimize the use of the current agents for the management of AMD. The role of other epigenetic factors, such as histone modification and noncoding RNA in the regulation of the pathogenesis of AMD is even more complicated, because these have multiple functions and their interactions with each other are complex. Mitochondria is a key organelle in the response to oxidative stress; recently a report from Atilano et al. showed that expression of *CFH* and *VEGF* and the DNA methylation profile are regulated by mitochondrial DNA variants; therefore, the role of the interaction between mitochondrial and epigenetic factors in the pathogenesis of AMD should be further explored [117].

Risk factors of AMD are complex, involving multiple genetic, environmental, and epigenetic aspects, such as DNA methylation, histone modification, noncoding RNA, and mRNA methylation, and interplays among these factors determine whether an individual will develop AMD (Fig. 5). Epigenetics is dynamically and constantly adapting to environmental changes. While studies of individual epigenetic factors that contribute to the pathogenesis of AMD are important, the interaction between genetics and epigenetics should also gain attention, given the significant interplay between SNP genotypes and CpG methylation. Importantly, increased TG levels are strongly associated with enhanced methylation at the revealed CpG sites [118]. Moreover, the genetic risk of rheumatoid arthritis may be mediated through DNA methylation [119]. To build a comprehensive map of the human epigenome, all updated information should be collated into a comprehensive analysis; furthermore, not only specimens from different populations and places are required, but these should also be collected at different time-points. Thus, as with EWAS, extensive international collaboration is needed.

The evidence of involvement of epigenetics in AMD pathogenesis may potentially lead to the use of epigenetics drugs for the treatment of AMD; however, studies of the use of epigenetic drugs, such as HDACis and DNMT, for AMD treatment in vivo are currently ongoing. A major problem in applying epigenetic agents for ocular disease is the lack of a target cell or target gene specificity. Another option for the treatment of AMD involves induction of stem cells to differentiate into RPE cells by epigenetic regulatory mechanisms and then replacing the dysfunctional RPE [120]. Recently, a synergistic inhibition of tumor growth was obtained by combining an HDAC inhibitor and a demethylase inhibitor [121]. Studies targeting a specific enzyme of the polycomb-repressor complex (EZH2 inhibitors) or acetylated lysine histone domains through bromodomain readers are considered as epigenetic therapy for cancer [122-124]. A systematic review may provide a clue for epigenetic research related to AMD. The task of the next decade will be to design specific drugs that can block the genetic and epigenetic alterations to prevent RPE from dysfunction or development of advanced AMD. Alternatively, it might be possible to combine the traditional therapy of AMD with epigenetic drugs to provide a new approach for the treatment of AMD.

## References

[1] Yonekawa Y, Miller JW, Kim IK (2015). Age-Related Macular Degeneration: Advances in Management and Diagnosis. J Clin Med, 4:343-359. 26239130 10.3390/jcm4020343 PMC4470128

[2] Meyers KJ, Liu Z, Millen AE, Iyengar SK, Blodi BA, Johnson E, et al (2015). Joint Associations of Diet, Lifestyle, and Genes with Age-Related Macular Degeneration. Ophthalmology, 122:2286-2294. 26354764 10.1016/j.ophtha.2015.07.029 PMC4714866

[3] van Lookeren Campagne M, LeCouter J, Yaspan BL, Ye W (2014). Mechanisms of age-related macular degeneration and therapeutic opportunities. J Pathol, 232:151-164. 24105633 10.1002/path.4266

[4] Chen Y, Bedell M, Zhang K (2010). Age-related macular degeneration: genetic and environmental factors of disease. Mol Interv, 10:271-281. 21045241 10.1124/mi.10.5.4 PMC3002218

[5] Mousavi M, Armstrong RA (2013). Genetic risk factors and age-related macular degeneration (AMD). Journal of Optometry, 6:176-184.

[6] Miyake M, Yamashiro K, Tamura H, Kumagai K, Saito M, Sugahara-Kuroda M, et al (2015). The Contribution of Genetic Architecture to the 10-Year Incidence of Age-Related Macular Degeneration in the Fellow Eye. Invest Ophthalmol Vis Sci, 56:5353-5361. 26275133 10.1167/iovs.14-16020

[7] Fritsche LG, Chen W, Schu M, Yaspan BL, Yu Y, Thorleifsson G, et al (2013). Seven new loci associated with age-related macular degeneration. Nat Genet, 45:433-439, 439e431-432. 23455636 10.1038/ng.2578 PMC3739472

[8] Thakkinstian A, Han P, McEvoy M, Smith W, Hoh J, Magnusson K, et al (2006). Systematic review and meta-analysis of the association between complement factor H Y402H polymorphisms and age-related macular degeneration. Hum Mol Genet, 15:2784-2790. 16905558 10.1093/hmg/ddl220

[9] Yasuma TR, Nakamura M, Nishiguchi KM, Kikuchi M, Kaneko H, Niwa T, et al (2010). Elevated C-reactive protein levels and ARMS2/HTRA1 gene variants in subjects without age-related macular degeneration. Mol Vis, 16:2923-2930. 21203342 PMC3013066

[10] Lin MK, Yang J, Hsu CW, Gore A, Bassuk AG, Brown LM, et al (2018). HTRA1, an age-related macular degeneration protease, processes extracellular matrix proteins EFEMP1 and TSP1. Aging Cell, 17: e12710. 29730901 10.1111/acel.12710 PMC6052470

[11] Millen AE, Meyers KJ, Liu Z, Engelman CD, Wallace RB, LeBlanc ES, et al (2015). Association between vitamin D status and age-related macular degeneration by genetic risk. JAMA Ophthalmol, 133:1171-1179. 26312598 10.1001/jamaophthalmol.2015.2715 PMC4841267

[12] Vladan B, Biljana SP, Mandusic V, Zorana M, Zivkovic L (2013). Instability in X chromosome inactivation patterns in AMD: a new risk factor? Med Hypothesis Discov Innov Ophthalmol, 2:74-82. 24600647 PMC3939760

[13] Yuan D, Yuan D, Liu X, Yuan S, Xie P, Liu Q (2013). Genetic association with response to intravitreal ranibizumab for neovascular age-related macular degeneration in the Han Chinese population. Ophthalmologica, 230:227-232. 24080590 10.1159/000355068

[14] Zhang S, Liu Y, Lu S, Cai X (2015). Genetic variants of interleukin 17A are functionally associated with increased risk of age-related macular degeneration. Inflammation, 38:658-663. 25028103 10.1007/s10753-014-9973-3

[15] Liang XY, Lai TY, Liu DT, Fan AH, Chen LJ, Tam PO, et al (2012). Differentiation of exudative age-related macular degeneration and polypoidal choroidal vasculopathy in the ARMS2/HTRA1 locus. Invest Ophthalmol Vis Sci, 53:3175-3182. 22491416 10.1167/iovs.11-8135

[16] Liang XY, Chen LJ, Ng TK, Tuo J, Gao JL, Tam PO, et al (2014). FPR1 interacts with CFH, HTRA1 and smoking in exudative age-related macular degeneration and polypoidal choroidal vasculopathy. Eye (Lond), 28:1502-1510. 25277308 10.1038/eye.2014.226 PMC4268466

[17] Liu K, Chen LJ, Tam PO, Shi Y, Lai TY, Liu DT, et al (2013). Associations of the C2-CFB-RDBP-SKIV2L locus with age-related macular degeneration and polypoidal choroidal vasculopathy. Ophthalmology, 120:837-843. 23260260 10.1016/j.ophtha.2012.10.003

[18] Zhang X, Li M, Wen F, Zuo C, Chen H, Wu K, et al (2013). Different impact of high-density lipoprotein-related genetic variants on polypoidal choroidal vasculopathy and neovascular age-related macular degeneration in a Chinese Han population. Exp Eye Res, 108:16-22. 23274582 10.1016/j.exer.2012.12.005

[19] Ji Y, Zhang X, Wu K, Su Y, Li M, Zuo C, et al (2014). Association of rs6982567 near GDF6 with neovascular age-related macular degeneration and polypoidal choroidal vasculopathy in a Han Chinese cohort. BMC Ophthalmol, 14:140. 25416513 10.1186/1471-2415-14-140 PMC4251681

[20] Yanagisawa S, Sakurada Y, Miki A, Matsumiya W, Imoto I, Honda S (2015). The association of elastin gene variants with two angiographic subtypes of polypoidal choroidal vasculopathy. PLoS One, 10: e0120643. 25775011 10.1371/journal.pone.0120643 PMC4361579

[21] SanGiovanni JP, Chew EY (2014). Clinical applications of age-related macular degeneration genetics. Cold Spring Harb Perspect Med, 4. 10.1101/cshperspect.a017228 PMC4200209 25125423

[22] Suuronen T, Nuutinen T, Ryhanen T, Kaarniranta K, Salminen A (2007). Epigenetic regulation of clusterin/apolipoprotein J expression in retinal pigment epithelial cells. Biochem Biophys Res Commun, 357:397-401. 17420006 10.1016/j.bbrc.2007.03.135

[23] Seddon JM, Reynolds R, Shah HR, Rosner B (2011). Smoking, dietary betaine, methionine, and vitamin D in monozygotic twins with discordant macular degeneration: epigenetic implications. Ophthalmology, 118:1386-1394. 21620475 10.1016/j.ophtha.2010.12.020 PMC3711586

[24] Hunter A, Spechler PA, Cwanger A, Song Y, Zhang Z, Ying G-s, et al (2012). DNA Methylation Is Associated with Altered Gene Expression in AMD. Investigative Opthalmology & Visual Science, 53:2089-2105. 10.1167/iovs.11-8449 PMC4108280 22410570

[25] Wei L, Liu B, Tuo J, Shen D, Chen P, Li Z, et al (2012). Hypomethylation of the IL17RC promoter associates with age-related macular degeneration. Cell Rep, 2:1151-1158. 23177625 10.1016/j.celrep.2012.10.013 PMC3513594

[26] Oliver VF, Jaffe AE, Song J, Wang G, Zhang P, Branham KE, et al (2015). Differential DNA methylation identified in the blood and retina of AMD patients. Epigenetics, 10:698-707. 26067391 10.1080/15592294.2015.1060388 PMC4622056

[27] Koks G, Uudelepp ML, Limbach M, Peterson P, Reimann E, Koks S (2015). Smoking-induced expression of the GPR15 gene indicates its potential role in chronic inflammatory pathologies. Am J Pathol, 185:2898-2906. 26348578 10.1016/j.ajpath.2015.07.006

[28] Arjamaa O, Nikinmaa M, Salminen A, Kaarniranta K (2009). Regulatory role of HIF-1alpha in the pathogenesis of age-related macular degeneration (AMD). Ageing Res Rev, 8:349-358. 19589398 10.1016/j.arr.2009.06.002

[29] Cascella R, Strafella C, Caputo V, Errichiello V, Zampatti S, Milano F, et al (2018). Towards the application of precision medicine in Age-Related Macular Degeneration. Prog Retin Eye Res, 63:132-146. 29197628 10.1016/j.preteyeres.2017.11.004

[30] Porter LF, Saptarshi N, Fang Y, Rathi S, den Hollander AI, de Jong EK, et al (2019). Whole-genome methylation profiling of the retinal pigment epithelium of individuals with age-related macular degeneration reveals differential methylation of the SKI, GTF2H4, and TNXB genes. Clin Epigenetics, 11:6.2-14 30642396 10.1186/s13148-019-0608-2 PMC6332695

[31] Kim MS, Kwon HJ, Lee YM, Baek JH, Jang JE, Lee SW, et al (2001). Histone deacetylases induce angiogenesis by negative regulation of tumor suppressor genes. Nat Med, 7:437-443. 11283670 10.1038/86507

[32] Ponnaluri VK, Vadlapatla RK, Vavilala DT, Pal D, Mitra AK, Mukherji M (2011). Hypoxia induced expression of histone lysine demethylases: implications in oxygen-dependent retinal neovascular diseases. Biochem Biophys Res Commun, 415:373-377. 22037463 10.1016/j.bbrc.2011.10.075

[33] To M, Yamamura S, Akashi K, Charron CE, Haruki K, Barnes PJ, et al (2012). Defect of adaptation to hypoxia in patients with COPD due to reduction of histone deacetylase 7. Chest, 141:1233-1242. 22172637 10.1378/chest.11-1536 PMC3342783

[34] Wang S, Li X, Parra M, Verdin E, Bassel-Duby R, Olson EN (2008). Control of endothelial cell proliferation and migration by VEGF signaling to histone deacetylase 7. Proc Natl Acad Sci U S A, 105:7738-7743. 18509061 10.1073/pnas.0802857105 PMC2409381

[35] Hsing CH, Hung SK, Chen YC, Wei TS, Sun DP, Wang JJ, et al (2015). Histone Deacetylase Inhibitor Trichostatin A Ameliorated Endotoxin-Induced Neuroinflammation and Cognitive Dysfunction. Mediators Inflamm, 2015:163140. 26273133 10.1155/2015/163140 PMC4530275

[36] Deroanne CF, Bonjean K, Servotte S, Devy L, Colige A, Clausse N, et al (2002). Histone deacetylases inhibitors as anti-angiogenic agents altering vascular endothelial growth factor signaling. Oncogene, 21:427-436. 11821955 10.1038/sj.onc.1205108

[37] Hrgovic I, Doll M, Pinter A, Kaufmann R, Kippenberger S, Meissner M (2017). Histone deacetylase inhibitors interfere with angiogenesis by decreasing endothelial VEGFR-2 protein half-life in part via a VE-cadherin-dependent mechanism. Exp Dermatol, 26:194-201. 27487811 10.1111/exd.13159

[38] Xing Y, Tu J, Zhang L, Guo L, Xi T (2015). Anti-angiogenic effect of tanshinone IIA involves inhibition of the VEGF/VEGFR2 pathway in vascular endothelial cells. Oncol Rep, 33:163-170. 25376085 10.3892/or.2014.3592

[39] https://patents.google.com/patent/WO2004043352A2 #patentCitations MXPA05004485A *2002-11-12 2005-1123- Alcon Inc Histone deacetylase inhibitors for the treatment of ocular neovascular or edematous disorders and diseases.

[40] Gnana-Prakasam JP, Veeranan-Karmegam R, Coothankandaswamy V, Reddy SK, Martin PM, Thangaraju M, et al (2013). Loss of Hfe leads to progression of tumor phenotype in primary retinal pigment epithelial cells. Invest Ophthalmol Vis Sci, 54:63-71. 23169885 10.1167/iovs.12-10312 PMC3544423

[41] Chan N, He S, Spee CK, Ishikawa K, Hinton DR (2015). Attenuation of choroidal neovascularization by histone deacetylase inhibitor. PLoS One, 10:e0120587. 25807249 10.1371/journal.pone.0120587 PMC4373846

[42] Crosson CE, Mani SK, Husain S, Alsarraf O, Menick DR (2010). Inhibition of histone deacetylase protects the retina from ischemic injury. Invest Ophthalmol Vis Sci, 51:3639-3645. 20164449 10.1167/iovs.09-4538 PMC2904015

[43] Poulose N, Raju R (2015). Sirtuin regulation in aging and injury. Biochim Biophys Acta, 1852:2442-2455. 26303641 10.1016/j.bbadis.2015.08.017 PMC4682052

[44] Tucci P (2012). Caloric restriction: is mammalian life extension linked to p53? Aging (Albany NY), 4:525-534. 22983298 10.18632/aging.100481 PMC3461340

[45] Kojiro Nakamura, Min Zhang, Shoichi Kageyama, et al (2017). Macrophage heme oxygenase-1-SIRT1-p53 axis regulates sterile inflammation in liver ischemia-reperfusion injury. J Hepatol, 67(6): 1232-1242. 28842295 10.1016/j.jhep.2017.08.010 PMC5884687

[46] Seo JS, Moon MH, Jeong JK, Seol JW, Lee YJ, Park BH, et al (2012). SIRT1, a histone deacetylase, regulates prion protein-induced neuronal cell death. Neurobiol Aging, 33:1110-1120. 21074897 10.1016/j.neurobiolaging.2010.09.019

[47] Vaziri H, Dessain SK, Ng Eaton E, Imai SI, Frye RA, Pandita TK, et al (2001). hSIR2(SIRT1) functions as an NAD-dependent p53 deacetylase. Cell, 107:149-159. 11672523 10.1016/s0092-8674(01)00527-x

[48] Brunet A, Sweeney LB, Sturgill JF, Chua KF, Greer PL, Lin Y, et al (2004). Stress-dependent regulation of FOXO transcription factors by the SIRT1 deacetylase. Science, 303:2011-2015. 14976264 10.1126/science.1094637

[49] Yeung F, Hoberg JE, Ramsey CS, Keller MD, Jones DR, Frye RA, et al (2004). Modulation of NF-kappaB-dependent transcription and cell survival by the SIRT1 deacetylase. Embo J, 23:2369-2380. 15152190 10.1038/sj.emboj.7600244 PMC423286

[50] Wang C, Chen L, Hou X, Li Z, Kabra N, Ma Y, et al (2006). Interactions between E2F1 and SirT1 regulate apoptotic response to DNA damage. Nat Cell Biol, 8:1025-1031. 16892051 10.1038/ncb1468

[51] Tan L, Yu JT, Guan HS (2008). Resveratrol exerts pharmacological preconditioning by activating PGC-1alpha. Med Hypotheses, 71:664-667. 18694626 10.1016/j.mehy.2008.06.031

[52] Lim JH, Lee YM, Chun YS, Chen J, Kim JE, Park JW (2010). Sirtuin 1 modulates cellular responses to hypoxia by deacetylating hypoxia-inducible factor 1alpha. Mol Cell, 38:864-878. 20620956 10.1016/j.molcel.2010.05.023

[53] Dioum EM, Chen R, Alexander MS, Zhang Q, Hogg RT, Gerard RD, et al (2009). Regulation of hypoxia-inducible factor 2alpha signaling by the stress-responsive deacetylase sirtuin 1. Science, 324:1289-1293. 19498162 10.1126/science.1169956

[54] Peng CH, Cherng JY, Chiou GY, Chen YC, Chien CH, Kao CL, et al (2011). Delivery of Oct4 and SirT1 with cationic polyurethanes-short branch PEI to aged retinal pigment epithelium. Biomaterials, 32:9077-9088. 21890195 10.1016/j.biomaterials.2011.08.008

[55] Maloney SC, Antecka E, Granner T, Fernandes B, Lim LA, Orellana ME, et al (2013). Expression of SIRT1 in choroidal neovascular membranes. Retina, 33:862-866. 23135526 10.1097/IAE.0b013e31826af556

[56] Zhuge CC, Xu JY, Zhang J, Li W, Li P, Li Z, et al (2014). Fullerenol protects retinal pigment epithelial cells from oxidative stress-induced premature senescence via activating SIRT1. Invest Ophthalmol Vis Sci, 55:4628-4638. 24845634 10.1167/iovs.13-13732

[57] Wei W, Li L, Zhang Y, Geriletu, Yang J, Zhang Y, et al (2014). Vitamin C protected human retinal pigmented epithelium from oxidant injury depending on regulating SIRT1. Scientific World Journal, 2014:750634. 25147862 10.1155/2014/750634 PMC4132313

[58] Li L, Wei W, Zhang Y, Tu G, Zhang Y, Yang J, et al (2015). SirT1 and STAT3 protect retinal pigmented epithelium cells against oxidative stress. Mol Med Rep, 12:2231-2238. 25847123 10.3892/mmr.2015.3570

[59] Richer S, Patel S, Sockanathan S, Ulanski LJ 2nd, Miller L, Podella C (2014). Resveratrol based oral nutritional supplement produces long-term beneficial effects on structure and visual function in human patients. Nutrients, 6:4404-4420. 25329968 10.3390/nu6104404 PMC4210925

[60] Turner RS, Thomas RG, Craft S, van Dyck CH, Mintzer J, Reynolds BA, et al (2015). A randomized, double-blind, placebo-controlled trial of resveratrol for Alzheimer disease. Neurology, 85:1383-1391. 26362286 10.1212/WNL.0000000000002035 PMC4626244

[61] Zhang H, He S, Spee C, Ishikawa K, Hinton DR (2015). SIRT1 mediated inhibition of VEGF/VEGFR2 signaling by Resveratrol and its relevance to choroidal neovascularization. Cytokine, 76:549-552. 26174951 10.1016/j.cyto.2015.06.019 PMC4605850

[62] Karali M, Persico M, Mutarelli M, Carissimo A, Pizzo M, Singh Marwah V, et al (2016). High-resolution analysis of the human retina miRNome reveals isomiR variations and novel microRNAs. Nucleic Acids Res, 44:1525-1540. 26819412 10.1093/nar/gkw039 PMC4770244

[63] Li Y, Mao L, Gao Y, Baral S, Zhou Y, Hu B (2015). MicroRNA-107 contributes to post-stroke angiogenesis by targeting Dicer-1. Sci Rep, 5:13316. 26294080 10.1038/srep13316 PMC4543985

[64] Askou AL, Alsing S, Holmgaard A, Bek T, Corydon TJ (2018). Dissecting microRNA dysregulation in age-related macular degeneration: new targets for eye gene therapy. Acta Ophthalmol, 96:9-23. 28271607 10.1111/aos.13407

[65] Lin H, Qian J, Castillo AC, Long B, Keyes KT, Chen G, et al (2011). Effect of miR-23 on oxidant-induced injury in human retinal pigment epithelial cells. Invest Ophthalmol Vis Sci, 52:6308-6314. 21693609 10.1167/iovs.10-6632

[66] Lukiw WJ, Surjyadipta B, Dua P, Alexandrov PN (2012). Common micro RNAs (miRNAs) target complement factor H (CFH) regulation in Alzheimer’s disease (AD) and in age-related macular degeneration (AMD). Int J Biochem Mol Biol, 3:105-116. 22509485 PMC3325769

[67] Sabatel C, Malvaux L, Bovy N, Deroanne C, Lambert V, Gonzalez ML, et al (2011). MicroRNA-21 exhibits antiangiogenic function by targeting RhoB expression in endothelial cells. PLoS One, 6:e16979. 21347332 10.1371/journal.pone.0016979 PMC3037403

[68] Kaneko H, Dridi S, Tarallo V, Gelfand BD, Fowler BJ, Cho WG, et al (2011). DICER1 deficit induces Alu RNA toxicity in age-related macular degeneration. Nature, 471:325-330. 21297615 10.1038/nature09830 PMC3077055

[69] Saxena K, Rutar MV, Provis JM, Natoli RC (2015). Identification of miRNAs in a Model of Retinal Degenerations. Invest Ophthalmol Vis Sci, 56:1820-1829. 25711632 10.1167/iovs.14-15449

[70] Strafella C, Errichiello V, Caputo V, Aloe G, Ricci F, Cusumano A, et al (2019). The Interplay between miRNA-Related Variants and Age-Related Macular Degeneration: EVIDENCE of Association of MIR146A and MIR27A. Int J Mol Sci, 20: pii: E1578 10.3390/ijms20071578 PMC6480223 30934838

[71] Lasser C (2012). Exosomal RNA as biomarkers and the therapeutic potential of exosome vectors. Expert Opin Biol Ther, 12 Suppl 1:S189-197. 22506888 10.1517/14712598.2012.680018

[72] Grassmann F, Schoenberger PG, Brandl C, Schick T, Hasler D, Meister G, et al (2014). A circulating microrna profile is associated with late-stage neovascular age-related macular degeneration. PLoS One, 9:e107461. 25203061 10.1371/journal.pone.0107461 PMC4159338

[73] Szemraj M, Bielecka-Kowalska A, Oszajca K, Krajewska M, Gos R, Jurowski P, et al (2015). Serum MicroRNAs as Potential Biomarkers of AMD. Med Sci Monit, 21:2734-2742. 26366973 10.12659/MSM.893697 PMC4576928

[74] Ren C, Liu Q, Wei Q, Cai W, He M, Du Y, et al (2017). Circulating miRNAs as Potential Biomarkers of Age-Related Macular Degeneration. Cell Physiol Biochem, 41:1413-1423. 28315863 10.1159/000467941

[75] Menard C, Rezende FA, Miloudi K, Wilson A, Tetreault N, Hardy P, et al (2016). MicroRNA signatures in vitreous humour and plasma of patients with exudative AMD. Oncotarget, 7:19171-19184. 27015561 10.18632/oncotarget.8280 PMC4991373

[76] Zhu W, Meng YF, Xing Q, Tao JJ, Lu J, Wu Y (2017). Identification of lncRNAs involved in biological regulation in early age-related macular degeneration. Int J Nanomedicine, 12:7589-7602. 29089757 10.2147/IJN.S140275 PMC5655033

[77] Chen X, Jiang C, Qin B, Liu G, Ji J, Sun X, et al (2017). LncRNA ZNF503-AS1 promotes RPE differentiation by downregulating ZNF503 expression. Cell Death Dis, 8:e3046. 28880276 10.1038/cddis.2017.382 PMC5636965

[78] Sivagurunathan S, Srikakulam N, Arunachalam JP, Pandi G, Chidambaram S (2018). In silico analysis of piRNAs in retina reveals potential targets in intracellular transport and retinal degeneration. bioRxiv:305144.

[79] Yang Y, Hsu PJ, Chen YS, Yang YG (2018). Dynamic transcriptomic m(6)A decoration: writers, erasers, readers and functions in RNA metabolism. Cell Res, 28:616-624. 29789545 10.1038/s41422-018-0040-8 PMC5993786

[80] Vu LP, Cheng Y, Kharas MG (2019). The Biology of m(6)A RNA Methylation in Normal and Malignant Hematopoiesis. Cancer Discov, 9:25-33. 30578356 10.1158/2159-8290.CD-18-0959

[81] Min KW, Zealy RW, Davila S, Fomin M, Cummings JC, Makowsky D, et al (2018). Profiling of m6A RNA modifications identified an age-associated regulation of AGO2 mRNA stability. Aging Cell, 17:e12753. 29573145 10.1111/acel.12753 PMC5946072

[82] Chang G, Leu JS, Ma L, Xie K, Huang S (2019). Methylation of RNA N(6)-methyladenosine in modulation of cytokine responses and tumorigenesis. Cytokine, 118:35-41. 30017390 10.1016/j.cyto.2018.06.018

[83] Chang G, Leu JS, Ma L, Xie K, Huang S (2019). Methylation of RNA N(6)-methyladenosine in modulation of cytokine responses and tumorigenesis. Cytokine, 118:35-41. 30017390 10.1016/j.cyto.2018.06.018

[84] Shen F, Huang W, Huang JT, Xiong J, Yang Y, Wu K, et al (2015). Decreased N(6)-methyladenosine in peripheral blood RNA from diabetic patients is associated with FTO expression rather than ALKBH5. J Clin Endocrinol Metab, 100:E148-154. 25303482 10.1210/jc.2014-1893 PMC5399497

[85] Huang H, Wang H, Zhou K, Wu T, Zhao BS, Sun M, et al (2019). Histone H3 trimethylation at lysine 36 guide m^6^A RNA modification co-transcriptionally. Nature, 567:414-419. 30867593 10.1038/s41586-019-1016-7 PMC6438714

[86] Clyde D (2019). Regulation of RNA methylation by modified histones. Nat Rev Genet, 20:254-255. 10.1038/s41576-019-0115-5 30890788

[87] Frank-Bertoncelj M, Klein K, Gay S (2017). Interplay between genetic and epigenetic mechanisms in rheumatoid arthritis. Epigenomics, 9:493-504. 28322583 10.2217/epi-2016-0142

[88] Hajjari M, Rahnama S (2019). Association Between SNPs of Long Non-coding RNA HOTAIR and Risk of Different Cancers. Front Genet, 10:113. 30873206 10.3389/fgene.2019.00113 PMC6403183

[89] Farh KK, Marson A, Zhu J, Kleinewietfeld M, Housley WJ, Beik S, et al (2015). Genetic and epigenetic fine mapping of causal autoimmune disease variants. Nature, 518:337-343. 25363779 10.1038/nature13835 PMC4336207

[90] Guo H, Ahmed M, Zhang F, Yao CQ, Li S, Liang Y, et al (2016). Modulation of long noncoding RNAs by risk SNPs underlying genetic predispositions to prostate cancer. Nat Genet, 48:1142-1150. 27526323 10.1038/ng.3637

[91] Lee SM, Kim-Ha J, Choi WY, Lee J, Kim D, Lee J, et al (2016). Interplay of genetic and epigenetic alterations in hepatocellular carcinoma. Epigenomics, 8:993-1005. 27411963 10.2217/epi-2016-0027

[92] Schulze K, Imbeaud S, Letouze E, Alexandrov LB, Calderaro J, Rebouissou S, et al (2015). Exome sequencing of hepatocellular carcinomas identifies new mutational signatures and potential therapeutic targets. Nat Genet, 47:505-511. 25822088 10.1038/ng.3252 PMC4587544

[93] Nishida N, Goel A (2011). Genetic and epigenetic signatures in human hepatocellular carcinoma: a systematic review. Curr Genomics, 12:130-137. 21966251 10.2174/138920211795564359 PMC3129047

[94] Tai DJ, Liu YC, Hsu WL, Ma YL, Cheng SJ, Liu SY, et al (2016). MeCP2 SUMOylation rescues Mecp2-mutant-induced behavioural deficits in a mouse model of Rett syndrome. Nat Commun, 7:10552. 26842955 10.1038/ncomms10552 PMC4743023

[95] Bannister AJ, Kouzarides T (2011). Regulation of chromatin by histone modifications. Cell Res, 21:381-395. 21321607 10.1038/cr.2011.22 PMC3193420

[96] anGiovanni JP, SanGiovanni PM, Sapieha P, De Guire V (2017). miRNAs, single nucleotide polymorphisms (SNPs) and age-related macular degeneration (AMD). Clin Chem Lab Med, 55:763-775. 28343170 10.1515/cclm-2016-0898

[97] Kim EJ, Grant GR, Bowman AS, Haider N, Gudiseva HV, Chavali VRM (2018). Complete Transcriptome Profiling of Normal and Age-Related Macular Degeneration Eye Tissues Reveals Dysregulation of Anti-Sense Transcription. Sci Rep, 8:3040. 29445097 10.1038/s41598-018-21104-7 PMC5813239

[98] Jones PA, Ohtani H, Chakravarthy A, De Carvalho DD (2019). Epigenetic therapy in immune-oncology. Nat Rev Cancer, 19:151-161. 30723290 10.1038/s41568-019-0109-9

[99] Moufarrij S, Dandapani M, Arthofer E, Gomez S, Srivastava A, Lopez-Acevedo M, et al (2019). Epigenetic therapy for ovarian cancer: promise and progress. Clinical Epigenetics, 11:7. 30646939 10.1186/s13148-018-0602-0 PMC6334391

[100] Kwa FA, Thrimawithana TR (2014). Epigenetic modifications as potential therapeutic targets in age-related macular degeneration and diabetic retinopathy. Drug Discov Today, 19:1387-1393. 24717156 10.1016/j.drudis.2014.03.026

[101] Maugeri A, Barchitta M, Mazzone MG, Giuliano F, Basile G, Agodi A (2018). Resveratrol Modulates SIRT1 and DNMT Functions and Restores LINE-1 Methylation Levels in ARPE-19 Cells under Oxidative Stress and Inflammation. Int J Mol Sci, 19. 10.3390/ijms19072118 PMC6073744 30037017

[102] He S, Barron E, Ishikawa K, Nazari Khanamiri H, Spee C, Zhou P, et al (2015). Inhibition of DNA Methylation and Methyl-CpG-Binding Protein 2 Suppresses RPE Transdifferentiation: Relevance to Proliferative Vitreoretinopathy. Invest Ophthalmol Vis Sci, 56:5579-5589. 26305530 10.1167/iovs.14-16258 PMC4553933

[103] Blanchard F, Chipoy C (2005). Histone deacetylase inhibitors: new drugs for the treatment of inflammatory diseases? Drug Discov Today, 10:197-204. 15708534 10.1016/S1359-6446(04)03309-4

[104] Bode KA, Schroder K, Hume DA, Ravasi T, Heeg K, Sweet MJ, et al (2007). Histone deacetylase inhibitors decrease Toll-like receptor-mediated activation of proinflammatory gene expression by impairing transcription factor recruitment. Immunology, 122:596-606. 17635610 10.1111/j.1365-2567.2007.02678.x PMC2266046

[105] Nencioni A, Beck J, Werth D, Grunebach F, Patrone F, Ballestrero A, et al (2007). Histone deacetylase inhibitors affect dendritic cell differentiation and immunogenicity. Clin Cancer Res, 13:3933-3941. 17606727 10.1158/1078-0432.CCR-06-2903

[106] Suraweera A, O’Byrne KJ, Richard DJ (2018). Combination Therapy With Histone Deacetylase Inhibitors (HDACi) for the Treatment of Cancer: Achieving the Full Therapeutic Potential of HDACi. Front Oncol, 8:92. 29651407 10.3389/fonc.2018.00092 PMC5884928

[107] Fox CR, Parks GD (2019). Histone Deacetylase Inhibitors Enhance Cell Killing and Block Interferon-Beta Synthesis Elicited by Infection with an Oncolytic Parainfluenza Virus. Viruses, 11:pii:E431. 10.3390/v11050431 PMC6563284 31083335

[108] Patnaik S, Anupriya (2019). Drugs Targeting Epigenetic Modifications and Plausible Therapeutic Strategies Against Colorectal Cancer. Front Pharmacol, 10:588. 31244652 10.3389/fphar.2019.00588 PMC6563763

[109] Nagai N, Kubota S, Tsubota K, Ozawa Y (2014). Resveratrol prevents the development of choroidal neovascularization by modulating AMP-activated protein kinase in macrophages and other cell types. J Nutr Biochem, 25:1218-1225. 25091551 10.1016/j.jnutbio.2014.05.015

[110] Ishida T, Yoshida T (2017). Potential role of sirtuin 1 in Muller glial cells in mice choroidal neovascularization. PLoS One, 12:e0183775. 28886036 10.1371/journal.pone.0183775 PMC5590853

[111] Chan N, He S, Spee CK, Ishikawa K, Hinton DR (2015). Attenuation of choroidal neovascularization by histone deacetylase inhibitor. PLoS One, 10:e0120587. 25807249 10.1371/journal.pone.0120587 PMC4373846

[112] Nagai N, Kubota S, Tsubota K, Ozawa Y (2014). Resveratrol prevents the development of choroidal neovascularization by modulating AMP-activated protein kinase in macrophages and other cell types. J Nutr Biochem, 25:1218-1225. 25091551 10.1016/j.jnutbio.2014.05.015

[113] Tanito M, Masutani H, Kim YC, Nishikawa M, Ohira A, Yodoi J (2005). Sulforaphane induces thioredoxin through the antioxidant-responsive element and attenuates retinal light damage in mice. Invest Ophthalmol Vis Sci, 46:979-987. 15728556 10.1167/iovs.04-1120

[114] Uchida H, Hayashi H, Kuroki M, Uno K, Yamada H, Yamashita Y, et al (2005). Vitamin A up-regulates the expression of thrombospondin-1 and pigment epithelium-derived factor in retinal pigment epithelial cells. Exp Eye Res, 80:23-30. 15652522 10.1016/j.exer.2004.08.004

[115] Feinberg AP (2010). Epigenomics reveals a functional genome anatomy and a new approach to common disease. Nat Biotechnol, 28:1049-1052. 20944596 10.1038/nbt1010-1049 PMC2956605

[116] Wang Y, Han Y, Fan E, Zhang K (2015). Analytical strategies used to identify the readers of histone modifications: A review. Anal Chim Acta, 891:32-42. 26388362 10.1016/j.aca.2015.06.049

[117] Atilano SR, Malik D, Chwa M, Caceres-Del-Carpio J, Nesburn AB, Boyer DS, et al (2015). Mitochondrial DNA variants can mediate methylation status of inflammation, angiogenesis and signaling genes. Hum Mol Genet, 24:4491-4503. 25964427 10.1093/hmg/ddv173 PMC4512622

[118] Fisher VA, Wang L, Deng X, Sarnowski C, Cupples LA, Liu CT (2018). Do changes in DNA methylation mediate or interact with SNP variation? A pharmacoepigenetic analysis. BMC Genet,19(Suppl 1):70. 30255765 10.1186/s12863-018-0635-6 PMC6156904

[119] Ventham NT, Kennedy NA, Adams AT, Kalla R, Heath S, O’Leary KR, et al (2016). Integrative epigenome-wide analysis demonstrates that DNA methylation may mediate genetic risk in inflammatory bowel disease. Nat Commun, 7:13507. 27886173 10.1038/ncomms13507 PMC5133631

[120] Binder S, Stanzel BV, Krebs I, Glittenberg C (2007). Transplantation of the RPE in AMD. Prog Retin Eye Res, 26:516-554. 17532250 10.1016/j.preteyeres.2007.02.002

[121] Mazur PK, Herner A, Mello SS, Wirth M, Hausmann S, Sanchez-Rivera FJ, et al (2015). Combined inhibition of BET family proteins and histone deacetylases as a potential epigenetics-based therapy for pancreatic ductal adenocarcinoma. Nat Med, 21:1163-1171. 26390243 10.1038/nm.3952 PMC4959788

[122] Cheng Z, Gong Y, Ma Y, Lu K, Lu X, Pierce LA, et al (2013). Inhibition of BET bromodomain targets genetically diverse glioblastoma. Clin Cancer Res, 19:1748-1759. 23403638 10.1158/1078-0432.CCR-12-3066 PMC4172367

[123] Lim DA, Alvarez-Buylla A (2014). Adult neural stem cells stake their ground. Trends Neurosci, 37:563-571. 25223700 10.1016/j.tins.2014.08.006 PMC4203324

[124] Yong RL, Tsankova NM (2015). Emerging interplay of genetics and epigenetics in gliomas: a new hope for targeted therapy. Semin Pediatr Neurol, 22:14-22. 25976256 10.1016/j.spen.2014.12.004

